# Assessment of soluble PD-L1 in septic shock in relation to immunosuppressive phenotypes

**DOI:** 10.1016/j.aicoj.2025.100007

**Published:** 2026-01-16

**Authors:** Camille Bonnet, Anne-Perrine Foray, Eléonore Micoud, Thomas Lafon, Morgane Gossez, Anne-Claire Lukaszewicz, Fabienne Venet, Guillaume Monneret

**Affiliations:** a Hospices Civils de Lyon, Immunology Laboratory, Hôpital E. Herriot and CH Lyon-Sud, Lyon France; b Emergency Department/Inserm CIC 1435, Limoges University Hospital, Limoges, France; c NLRP3 Inflammation and Immune Response to Sepsis Team, Centre International de Recherche in Infectiology (CIRI), Inserm U1111, CNRS, UMR5308, Ecole Normale Supérieure de Lyon, Claude Bernard University Lyon 1, Lyon France; d Hospices Civils de Lyon, Anesthesiology and Critical Care Medicine Department, Hôpital E. Herriot, Lyon, France; e Université de Lyon, EA 7426 “Pathophysiology of Injury-Induced Immunosuppression”, Université Claude Bernard Lyon^_^1, Lyon, France

**Keywords:** Septic shock, Hyperinflammation, Immunosuppression, Biomarkers

## Abstract

**Background:**

Septic shock triggers a complex immune response characterized by the coexistence of hyperinflammation and immunosuppression, the latter being a major driver of ICU-acquired infections and increased mortality. Currently, the most established biomarkers for assessing sepsis-induced immunosuppression rely on flow cytometry—a technique not universally available in clinical practice. In contrast, soluble biomarkers are, in principle, easier to measure. Although assays for soluble PD-L1 (sPD-L1) are not yet standardized, sPD-L1 concentrations may represent a pragmatic alternative, given the putative role of PD-1/PD-L1 signaling in immunosuppressive pathways during sepsis. In this study, we investigated sPD-L1 in relation to established cellular markers of immunosuppression in a cohort of 161 patients with septic shock. sPD-L1 levels were measured using the ELLA microfluidic platform during the first week of ICU admission. We assessed their association with clinical outcomes and explored the relationship between sPD-L1 and immunosuppressive profiles defined by low monocytic HLA-DR expression (mHLA-DR) and absolute lymphocyte count.

**Results:**

Upon admission, patients exhibited elevated sPD-L1 levels compared to healthy controls (medians: 179 vs. 54 pg/mL, p < 0.001). No correlation was observed between sPD-L1 levels and severity scores (SOFA, SAPS II). Elevated sPD-L1 was independently and significantly associated with increased mortality at both 28 and 90 days. Longitudinal analysis using K-means clustering revealed that the cluster with consistently highest sPD-L1 levels was associated with unfavorable outcomes. Overall, and at any single time point, sPD-L1 concentrations did not correlate with mHLA-DR expression or lymphopenia. However, the combined presence of high sPD-L1 and low mHLA-DR levels at the end of the first week identified a subgroup of patients with particularly poor clinical outcomes.

**Conclusions:**

These findings highlight the potential of sPD-L1 as a clinically relevant biomarker in the context of sepsis immunopathology. Further studies are warranted to elucidate its role in the mechanisms underlying sepsis-induced immunosuppression. Such insights could support the integration of sPD-L1 into multimodal biomarker panels for immune monitoring and risk stratification in patients with septic shock.

## Background

Sepsis is defined as life-threatening organ dysfunction resulting from a dysregulated host response to infection [1]. It is associated with high mortality, around 25%, increasing to 45–50 % in the most severely affected cases, such as septic shock [2]. It is responsible for nearly 20% of deaths worldwide, making it a global public health priority according to the World Health Organization [3].

The pathophysiology of sepsis is complex and dynamic, characterized by an intense initial hyperinflammatory response, accompanied by powerful compensatory mechanisms. In a substantial subset of patients who survive the initial hours, those anti-inflammatory feedbacks lead to a state of profound acquired immunosuppression. Classically, two distinct phases of mortality are described in a simplified model. The early phase, driven by hyperinflammation and organ failure, accounts for approximately 30% of sepsis-related deaths. The late phase, emerging after several days and potentially lasting for weeks, is associated with established immunosuppression, resulting in a higher risk of secondary infections and viral reactivation and accounting for about 70% of deaths [4,5]. While the mechanisms of hyperinflammation and associated therapeutic strategies have been extensively explored, the current challenge lies in better understanding sepsis-induced immunosuppression in order to develop targeted therapeutic approaches [6,7].

Septic patients exhibit both quantitative and qualitative alterations in innate and adaptive immune cells, resulting in global immunosuppression [5,8]. Among these alterations, T cell exhaustion—characterized by reduced proliferative capacity and cytokine secretion—has been reported. This exhaustion is notably associated with the overexpression of co-inhibitory molecules such as PD-1 [5]. Concurrently, an upregulation of its ligand, PD-L1, is observed on innate immune cells. Several studies have highlighted the role of the PD-1/PD-L1 pathway in sepsis pathophysiology [[9], [10], [11]] identifying it as a potential therapeutic target for immune restoration, similar to immunotherapy strategies used in oncology [4,11].

However, the significant variability in individual immune responses necessitates a personalized medicine approach. It is therefore crucial to identify the most immunosuppressed patients who are most likely to benefit from immunostimulatory treatments—especially since no clinical criteria currently allow for such identification [12,13]. In this context, biomarkers are essential. To date, the most commonly used biomarkers for assessing ICU-acquired immunosuppression (such as monocytic HLA-DR (mHLA-DR) expression, lymphopenia, and the percentage of immature neutrophils) mostly rely on flow cytometry [14]. Although highly informative, this technique requires skilled technical staff and laboratory infrastructure, limiting its accessibility. In contrast, soluble biomarkers are generally easier to measure and may represent a practical and promising alternative.

Within this framework, the soluble form of PD-L1 (sPD-L1) has emerged as a promising candidate biomarker. In oncology, several studies have demonstrated its immunosuppressive role as a PD-1 ligand [[15], [16], [17]]. In sepsis, studies have shown elevated sPD-L1 levels in patients compared to healthy volunteers, and preliminary data have indicated an association with disease severity and adverse outcomes [18,19]. Moreover, the potential of sPD-L1 as a biomarker of immunosuppression in sepsis is still underexplored. In particular, no study to date has assessed its correlation with the immunosuppression biomarkers commonly used in clinical practice.

Thus, the primary objective of the present work was to study the kinetics of sPD-L1 in septic shock patients and to evaluate its association with adverse outcomes, such as 28-day and 90-day mortality and the occurrence of ICU-acquired infections. The secondary objective was to investigate sPD-L1 within the context of sepsis-induced immunosuppression, by comparing it to established markers such as mHLA-DR expression and absolute lymphocyte count (ALC).

## Methods

### Patients and design

We included patients admitted to ICU in Anesthesiology and Intensive Care Department (Hôpital E. Herriot, Hospices Civils de Lyon) with septic shock (according to sepsis-3 definition). These patients belonged to the IMMUNOSEPSIS study registered at clinicaltrials.gov (NCT02803346 & NCT04067674). EDTA blood samples were obtained at day (D): D1-2, D3-4 and D5-8 (one sample per period). In addition, blood samples at 6 months after the septic shock were available for 7 patients. Because of the non interventional nature of the study, written informed consent was not required for inclusion. However, each clinician orally informed the patient or family members about the objectives, methodology, and conduct of the study, and provided them with a leaflet using plain language. Non opposition to participation to this study was systematically obtained from the patient or a third party before any blood sampling was performed and was recorded in patient’s clinical file. All procedures performed were in accordance with the ethical standards of the institutional and/or national research committee (Comité de Protection des Personnes Sud-Est II, IRB11236 and Comité de Protection des Personnes Sud-Ouest II, n° RCB: 2019-A00210-57, n°CPP: 19.01.23, 71857) and with the 1964 Helsinki declaration and its later amendments or comparable ethical standards. Clinical and biological databases were reported to the National Commission for Information Technology and Freedom (CNIL, number 08-27). A written non-opposition to the use of donated blood for research purposes was obtained from healthy volunteers (n = 50, median age = 34, M/F ratio = 17/33). Usual clinical data and biological data (age, sex, length of stay, type of infection, severity scores at admission (SAPS II, SOFA, Charlson), exposure to invasive devices, living or deceased status after 28 / 90 days, presence or absence of ICU acquired infection - as diagnosed in [20], mHLA-DR, absolute lymphocyte count) were collected.

The biological samples were stored in the Sepsis Biological Collection located at the Immunology Laboratory, Edouard Herriot Hospital, Hospices Civils de Lyon. The biological collection was recorded at the Ministry of Health (#DC-2008-509). Clinical and biological databases were reported to the Commission nationale de l’informatique et des libertés—National Commission for Information Technology and Freedom.

The inclusion criteria were patients admitted with septic shock and available plasma samples at D1-2, D3-4 and D5-8. The exclusion criteria were pregnant and breastfeeding women and any condition modifying the immune status: immunosuppressive treatment (including >10 mg equivalent prednisone per day or cumulative dose >700 mg), hematological disease treated within the 5 years, solid tumor treated with chemotherapy or in remission, number of circulating neutrophils less than 500/mm3, innate immune deficiency, and extracorporeal circulation within 1 month before inclusion (cardiopulmonary bypass or extracorporeal membrane oxygenation).

### Biomarkers measurement

sPD-L1 concentrations measurement was obtained with the ELLA microfluidic system (Bio-Techne, Minneapolis, USA), with the Simple Plex Kit - SPCKB-PS-003468 V5 (Human B7-H1 2nd gen/PD-L1). The measurements were carried out in triplicate (the mean of the 3 values was used). mHLA-DR expression and ALC were measured on whole blood with standardized flow cytometry. Regarding mHLA-DR expression, cells were marked with Quantibrite reagent (antibody and beads, Becton Dickinson, Franklin Lakes, NJ), as previously described [21]. Results were expressed in number of antibodies bound to cell (AB/C). Immunosuppressed status at D5-8 was defined when patients presented with both mHLA-DR < 8,000 AB/C [20] and ALC < 0.9 G/L [22].

### Statistical analysis

Statistical analyses were conducted using R software (version 4.4.2; Boston, MA, USA) and RStudio (version 2024.04.1; Posit Software, PBC). The normality of data distributions was assessed using the Shapiro–Wilk test. For comparisons between two groups, parametric or non-parametric tests were applied depending on the distribution: Student’s t-test was used for normally distributed quantitative variables, and the Mann–Whitney U test was employed otherwise. Qualitative variables were compared using the Chi-square test.

The temporal evolution of biological parameters was analyzed using the Friedman test, followed by Conover’s post hoc test for multiple comparisons. Comparisons between healthy volunteers and patient subgroups at different time points were performed using the Kruskal-Wallis test, with Dunn’s test applied for post hoc pairwise analysis. Receiver Operating Characteristic (ROC) curves were used to determine the area under the curve (AUC) for selected parameters. Survival analyses were performed using the Kaplan-Meier method, and differences between survival curves were evaluated with the log-rank test. Univariate analyses were conducted based on median stratification of each parameter. Parameters with a statistically significant odds ratio in univariate analysis were subsequently included in multivariate analyses. Correlations between biological markers were assessed using Spearman’s rank correlation coefficient. Finally, patient phenotypes were identified based on the longitudinal trajectories of sPD-L1 concentrations, using a supervised trajectory clustering approach derived from the K-means algorithm [23]. Overall, results are presented as median and [Q1-Q3].

## Results

### Overall cohort description

We included 161 patients with septic shock between March 2014 and March 2019. The main clinical characteristics are summarized in Table 1. The cohort was predominantly male (71%), with a median age of 67 years [55–76]. The median SOFA score was 9 [8–11], the median SAPS II score was 61 [49–71], and the median Charlson Comorbidity Index was 2 [1–4]. The 28-day mortality rate was 24 %, increasing to 32 % at 90 days. ICU-acquired infections in 28% of patients. The median ICU length of stay was 14 days [9–24]. The values for mHLA-DR and absolute lymphocyte count (ALC) at each time point are detailed in Table 2, highlighting patients with pronounced decrease in mHLA-DR expression and persistent lymphopenia. Overall, the clinical and immunological profiles observed in this cohort are consistent with those typically reported in septic shock and reflect a high level of disease severity. We next assessed the clinical and immunological characteristics of the cohort according to 28-day mortality, 90-day mortality and ICU-acquired infections occurrence. Result are depicted in Table 2.

**Table 1:** Patients’ main characteristics. Results are expressed as % or median [Q1-Q3].

Patients’ characteristics	
Sex (% of men)	70.8
Age	67 [55−76]
Severity scores at admission	
SOFA score	9 [8–11]
SAPS2 score	61 [49−71.25]
Charlson score	2 [1–4]
Exposure to invasive devices	
Duration of mechanical ventilation (days)	8 [4–15]
Duration of central venous catheterization (days)	12 [8–21]
Duration of urinary catheterization (days)	12 [8–22]
Primary site of infection	
Respiratory (%)	28.6
Intra-abdominal (%)	39.1
Other origins (%)	31.7
Adverse outcomes	
28-day mortality (%)	24.2
90-day mortality (%)	31.7
ICU acquired infections (%)	28.0
Duration of ICU stay (days)	14 [9–24]
Immunological parameters	
mHLA-DR D1-2	3,534 [2,462−5,734]
mHLA-DR D3-4	3,959 [2,050−6,360]
mHLA-DR D5-8	6,426 [3,540−10,059]
ALC D1-2	0.9 [0.6−1.4]
ALC D3-4	0.9 [0.6−1.2]
ALC D5-8	1.1 [0.8−1.6]

mHLA-DR results are expressed as AB/C (antibody bound / cell). Absolute lymphocyte count (ALC) results are expressed as number of cells (G/L).

**Table 2:** Clinical characteristics according to 28-day mortality, 90-day mortality and ICU-acquired infections.

	28-day mortality			90-day mortality			ICU acquired infections		
	Survivors	Non-survivors	*p-*value	Survivors	Non-survivors	*p-*value	Nosocomial infection	No infection	*p-*value
	122	39		110	51		45	116	
Genre (% of men)	69.7	74.4	0.575	69.1	74.5	0.481	80	67.2	0.11
Age	67 [55−76]	67 [59−77]	0.771	67 [55−76]	69 [59−77]	0.671	64 [52−72]	69 [59−77]	0.056
Severity scores at admission									
SOFA score	9 [8–11]	10 [8–12]	0.523	9 [8–11]	9 [8–11]	0.695	10 [7–12]	9 [8–11]	0.283
SAPS II score	60 [48−69]	64 [56−78]	0.068	60 [48−69]	64 [54−77]	0.166	61 [52−73]	62 [48−71]	0.727
Charlson comorbidity index	2 [1−3.8]	3 [2–4]	**< 0.01**	2 [0−3]	3 [2–4]	**< 0.001**	2 [1−3.3]	2 [1–4]	0.467
Exposure to invasive devices									
Duration of mechanical ventilation (days)	7 [4–14]	11 [8–17]	< 0.05	7 [4–14]	10 [6–17]	0.068	17 [10–24]	6 [3–10]	**< 0.001**
Duration of central venous catheterization (days)	12 [7–22]	12 [10–18]	0.606	11 [7–20]	15 [10–23]	**< 0.05**	22 [16−35]	10 [7–16]	**< 0.001**
Duration of urinary catheterization (days)	12 [7–24]	13 [10–17]	0.914	11 [7–22]	15 [10–20]	0.221	23.5 [15−38]	11 [7–16]	**< 0.001**
Immunological parameters									
mHLA-DR D1−2	3,451 [2,371−5,550]	4,025 [2,575–5,830]	0.386	3,456 [2,371−5,408]	3,970 [2,570−5,946]	0.270	3,300 [2,015−5,660]	3,649 [2,466−5,750]	0.385
mHLA-DR D3-4	3,890 [2,204-6,745]	4,307 [1,963–5494]	0.56	3,800 [2,083–6,626]	4,140.5 [2,185–5,978]	0.902	3,146 [1,810–5,172]	4,140 [2,527–6,636]	0.056
mHLA-DR D5−8	6,811 [4,417−10,137]	4,865 [2,428−8,976]	**< 0.05**	7,021 [4,162−10,131]	5,237 [3,119−9,209]	0.085	5028 [3,156−7,033]	7,273 [4,281−11,126]	**< 0.01**
ALC D1−2	0.9 [0.6−1.4]	1 [0.7−1.4]	0.804	0.9 [0.6−1.4]	1.1 [0.7−1.4]	0.485	0.8 [0.6−1.1]	1 [0.6−1.6]	0.11
ALC D3−4	1 [0.6−1.3]	0.8 [0.5–1]	0.072	1 [0.6−1.2]	0.8 [0.5−1.1]	0.144	0.8 [0.6−1.1]	0.9 [0.6−1.1]	0.324
ALC D5−8	1.2 [0.9−1.7]	1 [0.6–1.2]	**< 0.05**	1 [0.9–1.7]	1 [0.7–1.3]	**< 0.05**	1 [0.9–1.4]	1.1 [0.8−1.7]	0.515

Results are expressed as % or median [Q1-Q3]. mHLA-DR results are expressed as AB/C (antibody bound / cell). Absolute lymphocyte count (ALC) results are expressed as number of cells (G/L). Variables were compared using Mann–Whitney and Chi2 tests.

### sPD-L1 in septic shock

**Fig. 1:**
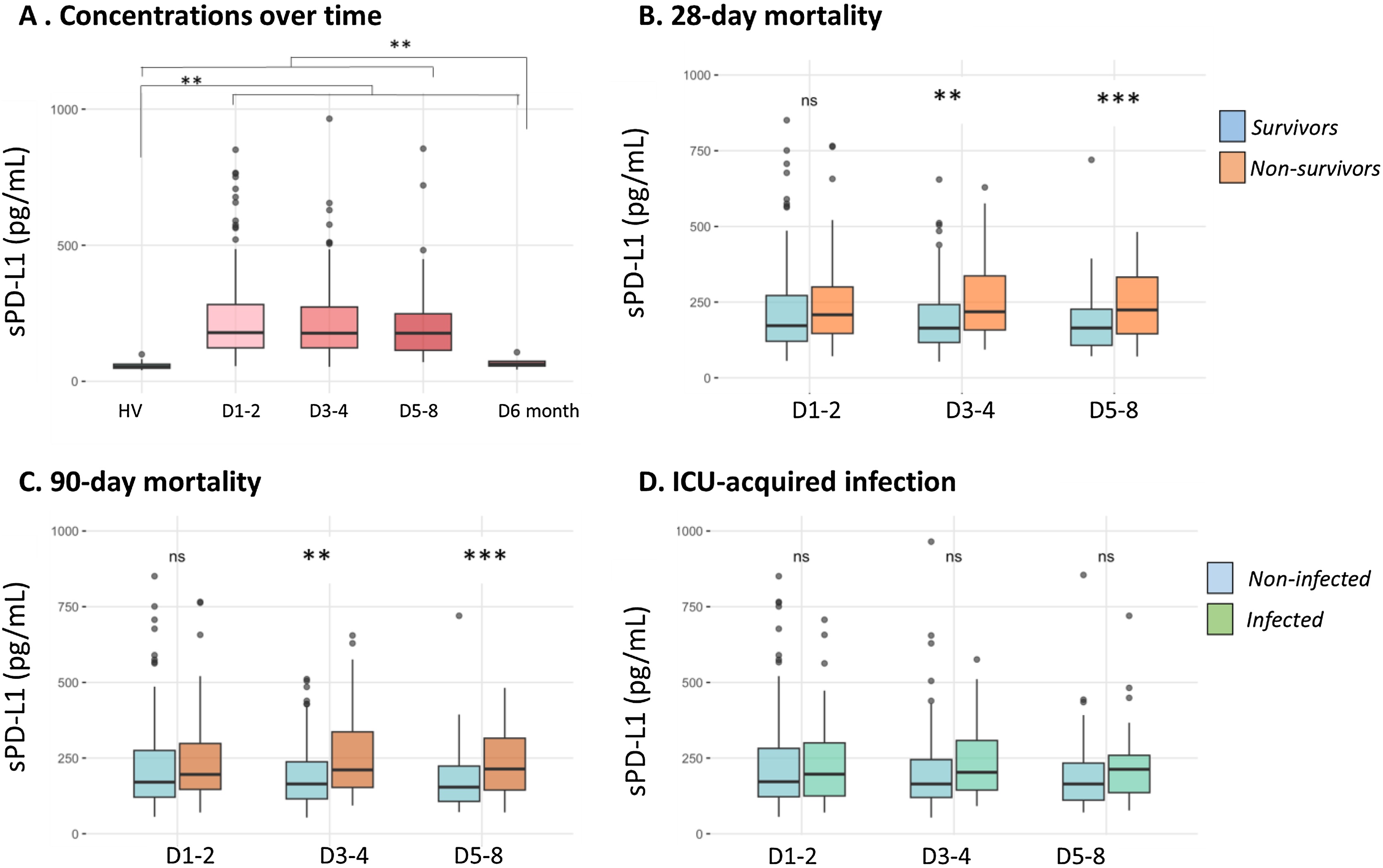
Temporal Dynamics of Soluble PD-L1 in Septic Shock: Analysis in the Whole Cohort and Stratification by Clinical Outcome. (A) sPD-L1 concentration in septic shock patients compared to healthy volunteers (HV) and its evolution at D1-2, D3-4, D5-8 and 6 months. (B) sPD-L1 concentrations comparison between 28-day survivors (n = 122) and non-survivors (n = 39) at D1-2, D3-4, D5-8. (C) sPD-L1 concentrations comparison between 90-day survivors (n = 110) and non-survivors (n = 45) at D1-2, D3-4, D5-8. (D) sPD-L1 concentrations comparison between patients with (n = 45) and without ICU-acquired infection (n = 116) at D1-2, D3-4, D5-8. sPD-L1 values were censored once infections occurred. Comparison of each with Mann-Whitney tests = ns: non significant; * p-value < 0,05; ** p-value < 0.01; *** p-value < 0.001.

sPD-L1 concentrations are represented in Fig. 1A. They were significantly increased in septic patients compared to healthy volunteers (medians: 179, 177, 177 pg/mL at D1-2, D3-4, D5-8 respectively vs HV: 54 pg/mL, p < 0.0001). Of note, the healthy control cohort was not age-matched; however, the values observed are consistent with those reported in previous studies [19,[24], [25], [26]]. Concentrations remained stable during the first week and no difference between the 3 time points. When available (n = 7), 6-month sPD-L1 returned to reference levels and were found not statistically different from the concentration of healthy volunteers. At day 1-2, we did not find any significant correlation between sPD-L1 and severity scores (SOFA, SAPS II, Charlson, data not shown). We next assessed sPD-L1 concentrations in relation to the three main adverse outcomes: 28-day mortality, 90-day mortality, and the occurrence of ICU-acquired infections, as shown in Fig. 1B–D. While sPD-L1 concentrations at D1–2 did not differ between survivors and non-survivors (at either 28 or 90 days), significant differences were observed at D3–4 and D5–8 (Fig. 1B-C). In contrast, no significant association was found between sPD-L1 levels and the occurrence of ICU-acquired infections at any time point (Fig. 1D).

### sPD-L1 and mortality in septic shock

**Fig. 2:**
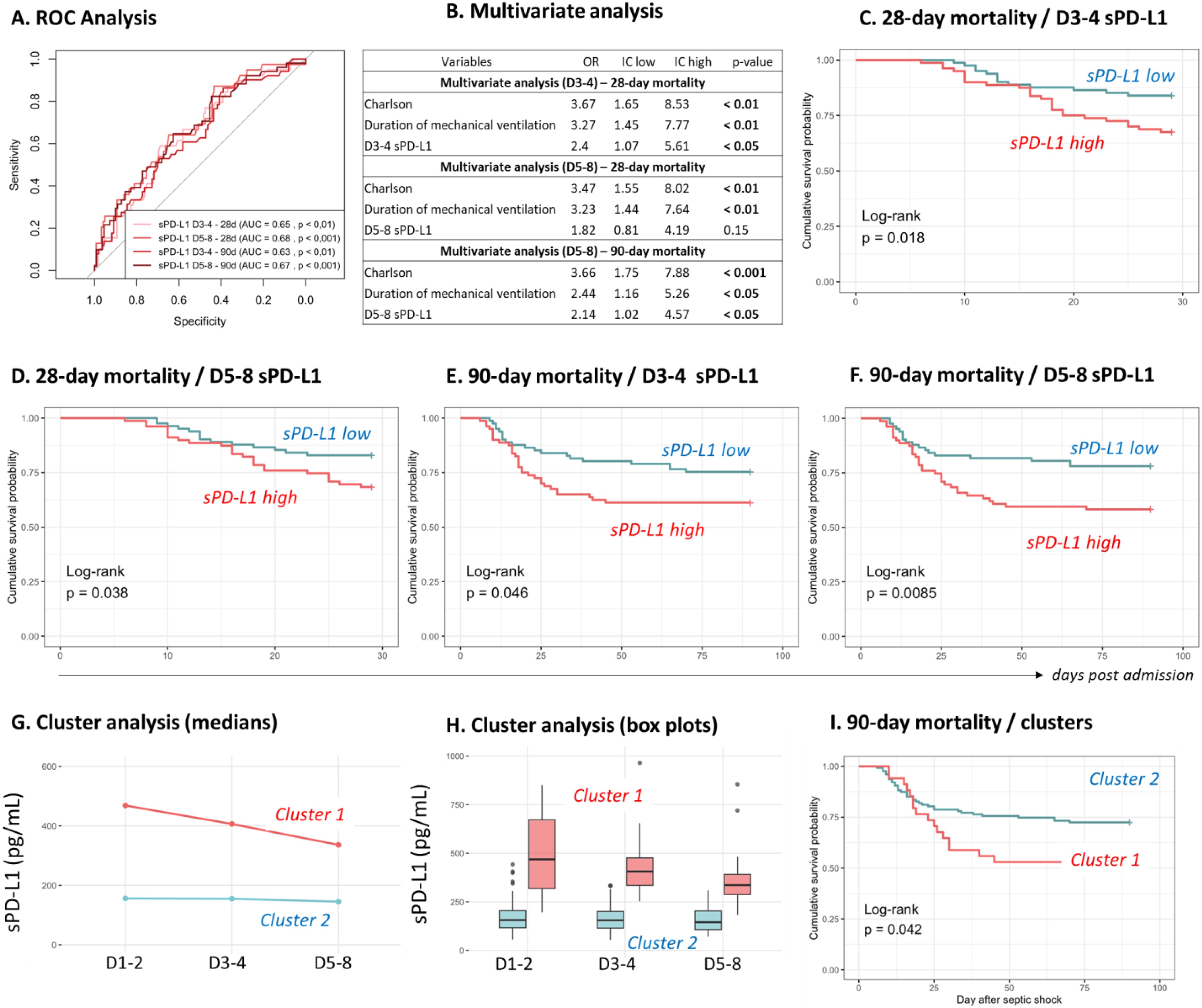
sPD-L1 association with mortality and cluster analysis. (A) ROC curves to predict 28- and 90-day mortality based on D3-4 or D5-8 sPD-L1 concentrations. (B) Table of multivariate analysis to predict 28- and 90-day mortality. Only the parameters significant in univariate analysis were included. (C) Kaplan Meier of 28-day mortality, stratification on D3-4 median sPD-L1, with high sPD-L1 and low sPD-L1. (D) Kaplan Meier of 28-day mortality, stratification on D5-8 median sPD-L1, with high sPD-L1 and low sPD-L1. (E) Kaplan Meier of 90-day mortality, stratification on D3-4 median sPD-L1, with high sPD-L1 and low sPD-L1. (F) Kaplan–Meier of 90-day mortality, stratification on D5-8 median sPD-L1, with high sPD-L1 and low sPD-L1. The log rank test was used to test the difference between the curves. (G) Median sPD-L1 trajectories according to the clusters obtained with K-means approach, cluster 1 (n = 127) and cluster 2 (n = 34). **(H)** Box plot presentation of sPD-L1 concentrations according to both clusters at different time-points. (I) Kaplan–Meier curves depicting 90-day mortality according to cluster assignment.

Based on previous results, we further explored the association between sPD-L1 levels and mortality specifically at D3–4 and D5–8. The area under curve (AUC) for each condition was significantly found from 0.63 to 0.67 (Fig. 2A). We then performed univariate analysis to predict 28- and 90-day mortality. Statistically significant parameters were the Charlson comorbidity index (OR = 3.27 [1.55−7.11], p < 0.01), the duration of mechanical ventilation (OR = 3.05 [1.43–6.82], p < 0.01) and sPD-L1 concentrations at D3−4 (OR = 2.52 [1.2–5.95], p < 0.05) and D5-8 (OR = 2.25 [1.08–4.84], p < 0.05). All of them were then integrated to the multivariate analysis (Fig. 2B). sPD-L1 concentrations at D3-4 (OR = 2.4 [1.07–5.61], p < 0.05) was independently associated with 28-day mortality whereas sPD-L1 concentrations at D5-8 was independently associated with 90-day mortality (OR = 2.14 [1.02–4.57], p < 0.05). Kaplan Meier curves illustrated and agreed with those results (Fig. 2C–F). Next, due to the limited sample size, a supervised K-means clustering restricted to two clusters was performed. We identified two clusters characterized by distinct sPD-L1 trajectories. The larger cluster (n = 127) exhibited stable, lower sPD-L1 levels over time, whereas the smaller cluster (n = 34) was marked by the highest levels, which declined only slowly over time (Fig. 2G-H). The main clinical characteristics of these clusters are presented in Table 3. Patients in the cluster with higher sPD-L1 concentrations had significantly higher SOFA scores, increased 90-day mortality, and longer ICU stays (Table 3 and Fig. 2I).

**Table 3:** Main clinical characteristics and outcomes of the 2 clusters of longitudinal sPD-L1 values from K-means analysis.

	Cluster 1 (n = 127)	Cluster 2 (n = 34)	*p*-value
Genre (% of men)	70.8	70.6	0.974
Age	68 [55−76]	63 [55.5−73.5]	0.343
Severity scores at admission			
SOFA score	9 [7–11]	10 [9−12.75]	**< 0.01**
SAPS II score	59.5 [48−69.75]	63 [54−76.75]	0.127
Charlson comorbidity index	2 [1–3]	3 [1–4]	0.278
Adverse outcomes			
28-day mortality (% of death)	21.3	35.3	0.089
90-day mortality (% of death)	27.6	47.1	**< 0.05**
ICU acquired infections (%)	25.9	35.3	0.282
Duration of ICU stay (days)	13 [9–23]	21 [14–28]	**< 0.01**
Immunosuppression status at D5-8 (yes, %)	23.6	32	0.4

Results are expressed as % or median [Q1-Q3]. Variables were compared using Mann–Whitney and Chi^2^ tests. Immunosuppressed status at D5-8 was defined when patients presented with both mHLA-DR < 8,000 AB/C (20) and ALC < 0.9 G/L (22).

### Association of sPD-L1 with usual immunosuppression markers

**Fig. 3:**
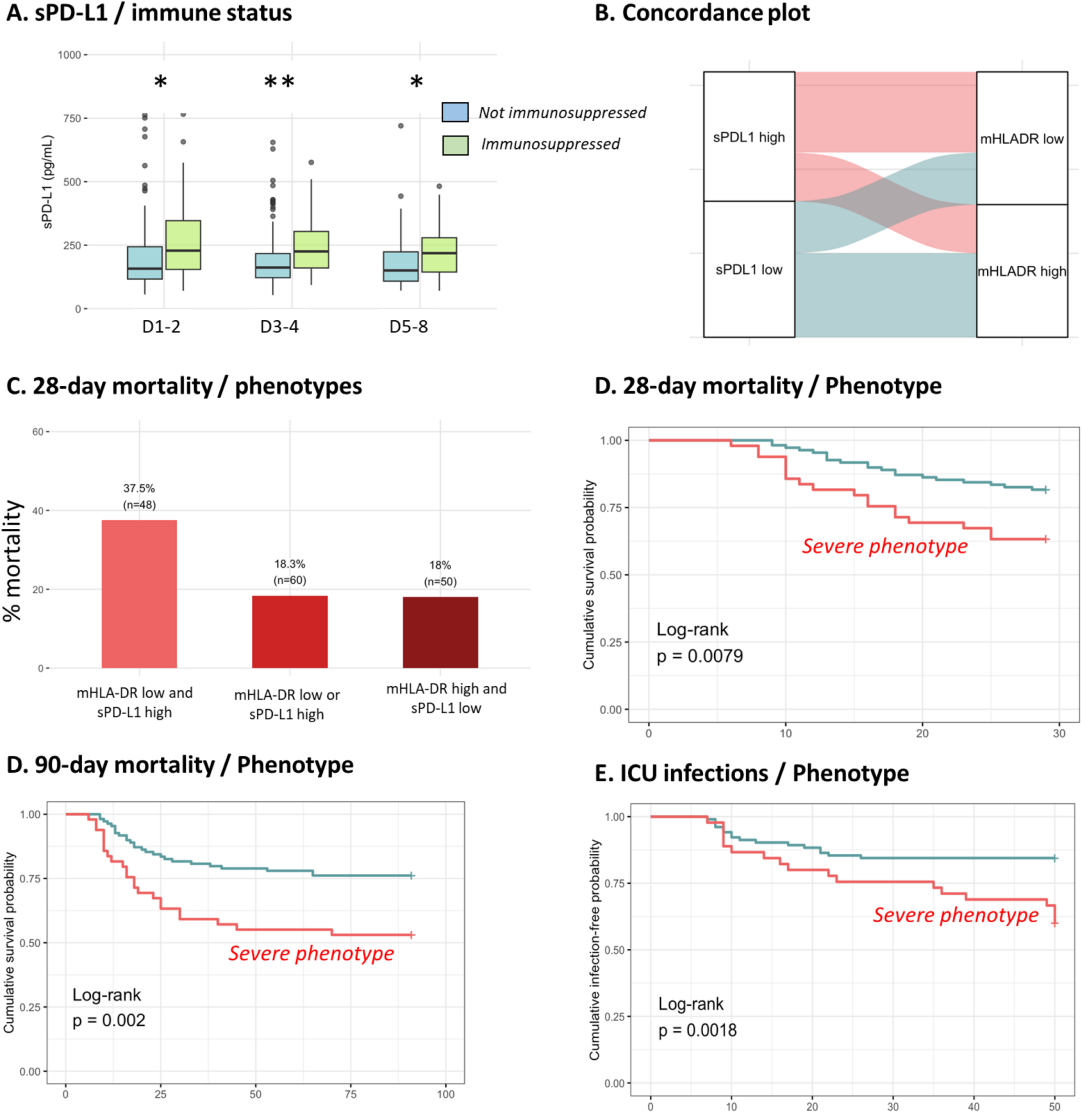
sPD-L1 and mHLA-DR combined can predict adverse outcomes in septic shock patients. (A) sPD-L1 concentrations comparison between immunosuppressed (n = 36) and non immunosuppressed patients (n = 106). Comparison of each populations at different time-points with Mann-Whitney test = * p-value < 0.05 ; ** p-value < 0.01. Immunosuppressed status at D5-8 was defined when patients presented with both mHLA-DR < 8,000 AB/C (20) and ALC < 0.9 G/L (22). (B) Alluvial plot to compare two groups of patients with high or low sPD-L1 stratified on D5-8 median and two groups of patients with high or low mHLA-DR stratified on D5-8 median. (C) Percentage of 28-day mortality according to different categories of patients: severe patients with low mHLA-DR and high sPD-L1; patients with either low mHLA-DR or high sPD-L1 and non-severe patients with high mHLA-DR and low sPD-L1. (D) Kaplan–Meier analysis for 28-day mortality, between severe patients with low mHLA-DR and high sPD-L1 (n = 49) and the rest of the cohort (n = 109). (E) Kaplan Meier analysis for 90-day mortality, between severe patients with low mHLA-DR and high sPD-L1 (n = 49) and the rest of the cohort (n = 109). (F) Kaplan–Meier of ICU-acquired infections (% cumulative infection free), between severe patients with low mHLA-DR and high sPD-L1 (n = 45) and the rest of the cohort (n = 103). The log rank test was used to test the difference between the curves.

We next investigated association of sPD-L1 with mHLA-DR and ALC. We first correlated parameters obtained at each time points. Except a significant but very slight correlation (r = 0.15, P < 0.05) with mHLA-DR at D1-2, we did not observe any relevant and significant correlation (data not shown). We then defined an immunosuppressed status at D5-8 when patients presented with both mHLA-DR < 8,000 AB/C and ALC < 0.9 G/L. Among 142 patients for whom those 2 parameters were available, 36 patients (25 %) felt in this immunosuppressed category. Main clinical characteristics are depicted in Table 4. The 28-day mortality was higher in immunosuppressed patients (36 % versus 19%, p < 0.05). Interstingly, Immunosuppressed patients had significant higher sPD-L1 concentrations at eachtimepoints (Fig. 3A). Using an alluvial plot, we next assessed the concordance between sPD-L1 and mHLA-DR levels at D5–8, by arbitrarily stratifying patients based on the median values of each marker. Concordance was observed in 62% of cases, consistent with the lack of a strong correlation between the two parameters (Fig. 3B). Nonetheless, we hypothesized that the combination of low mHLA-DR expression and high sPD-L1 concentrations could be associated with poor prognosis. Indeed, 28-day mortality reached 38% in patients exhibiting both biomarker abnormalities, compared to approximately 18% in those with only one or neither alteration (Fig. 3C). We illustrated this using Kaplan-Meier curves (Fig. 3D–F), which show that the group with low mHLA-DR and high sPD-L1 at days 5–8 was significantly associated with both 28-day (p = 0.008) and 90-day mortality (p = 0.002), as well as an increased rate of ICU-acquired infections (p = 0.002).

**Table 4:** Main clinical characteristics according to immunosuppression status established at D5-8.

	Immunosuppression (n = 36)	No immunosuppression (n = 106)	p-value
Genre (% of men)	86.11	65.09	**< 0.05**
Age	63 [54.75−71]	68 [59.25−76.75]	**< 0.05**
Severity scores at admission			
SOFA score	10 [9−11.25]	9 [7–11]	0.169
SAPS II score	59 [51.50–66.50]	61 [47−70.75]	0.374
Charlson comorbidity index	2 [0.75−4]	2 [1–4]	0.760
Adverse outcomes			
28-day mortality (% of death)	36	19	**< 0.05**
90-day mortality (% of death)	42	26	0.085
ICU acquired infections (%)	36	24	0.142
Duration of ICU stay (days)	18 [10–24]	14 [9–25]	0.276
PD-L1 (pg/mL)			
D1-2	229	158	**< 0.05**
D3-4	226	162	**< 0.05**
D5-7	219	151	**< 0.05**

Results are expressed as % or median [Q1-Q3]. Variables were compared using Mann-Whitney and Chi2 tests.

## Discussion

In this study, we investigated sPD-L1 concentration evolution in septic shock patients during the first week after ICU admission and its association with adverse outcomes. A few teams had already explored different aspect of sPD-L1 in sepsis. However, to date, no study specifically investigated sPD-L1 in a septic shock cohort and most importantly no work has been conducted within the context of sepis-induced immunosuppression.

First, and unsurprisingly, our results demonstrated an increase in sPD-L1 levels in septic shock patients compared to healthy volunteers. This finding aligns with previous studies conducted in various septic conditions [18,19,24,27,28]. However, due to the use of different measurement techniques across studies, direct comparison of absolute values remains difficult. Still, most studies report an approximate 3.5-fold increase in septic patients relative to healthy controls. We also observed that these elevated sPD-L1 concentrations remained remarkably stable during the first week following the onset of septic shock. Importantly, dynamic K-means clustering revealed that the subgroup of patients who consistently exhibited the highest sPD-L1 levels throughout the monitoring period had a striking 90-day mortality rate of 47%. This persistent elevation may be due either to sustained production of sPD-L1 during the first week of sepsis or to a prolonged half-life of the molecule. As no data on sPD-L1 half-life are currently available in the literature, this aspect warrants further investigation. All the more so since the study by Yende et al. [29] reports a sustained elevation of sPD-L1 lasting up to one year.

We did not find any correlation between admission severity scores and sPD-L1 concentrations, whereas Liu et al. and Kawamoto et al. reported an association with the SOFA score [27,30]. This discrepancy may be explained by differences in patient populations: in Liu’s study, the median SOFA score was relatively low, and in Kawamoto’s study, the sample size was small (n = 27). Nonetheless, despite the lack of correlation with severity scores, we demonstrated that sPD-L1 levels were significantly associated with both 28-day and 90-day mortality, even after multivariate analysis, indicating that elevated sPD-L1 is an independent predictor of mortality. These findings are consistent with those of Liu et al. [27] and Derigs et al. [26].

The most significant contribution of the present study is the investigation of sPD-L1 in the context of sepsis-induced immunosuppression—an aspect that, to our knowledge, has not previously been explored at the cellular level. Overall, we did not observe a correlation between sPD-L1 and established biomarkers of immunosuppression such as mHLA-DR or ALC. This was also illustrated by the concordance diagram, which showed no strong agreement between low mHLA-DR values and high sPD-L1 concentrations. However, when defining an "immunosuppressed" status as the presence of both mHLA-DR < 8,000 AB/C and ALC < 0.9 G/L at days 5–8, we found that these patients consistently exhibited higher sPD-L1 levels throughout the monitoring period. This supports the hypothesis that immunosuppression is associated with broader disruptions of immune homeostasis. Thus, combining low mHLA-DR and high sPD-L1 at days 5–8 identifies severely ill patients with increased mortality and ICU-acquired infections, only partially captured by mHLA-DR alone. While sPD-L1 cannot yet be classified as a conventional immunosuppressive biomarker, it nonetheless emerges as a promising candidate for identifying patients at higher risk of immune-inflammatory dysregulation after one week in the ICU. The putative biological / pathological role of sPD-L1 remains to be fully investigated. Indeed, in cancer, results showed contradictory results. Some suggested an immunosuppressive role [15–17] whereas other demonstrated an antagonist role of sPD-L1 [31].

Our study has several limitations. We included only septic shock patients for whom biological samples were available at all three time points (D1–2, D3–4, and D5–8). As a result, the cohort consisted of a specific subset of severe patients who survived at least until days 5–8. Patients with the most intense inflammatory responses—those who may have died within the first few days—were therefore not represented in this study. This selection may have led to a smoothing of the variability typically associated with sepsis severity, potentially influencing the observed immunological profiles. Additionally, we did not compare soluble PD-L1 (sPD-L1) levels with the membrane expression of PD-L1 and PD-1 on immune cells. Such data could have provided valuable insights for interpreting our findings and understanding potential links with immunosuppression. This aspect warrants further investigation in dedicated prospective studies allowing for real-time immune cell staining in whole blood.

## Conclusions

We observed increased sPD-L1 concentrations in septic shock patients compared to healthy volunteers. Elevated sPD-L1 levels were significantly associated with both 28-day and 90-day mortality, independently of clinical confounding factors. Consistently, clustering of sPD-L1 trajectories revealed a clear association between persistently high sPD-L1 levels and poor outcomes. While no strong associations were found between sPD-L1 and conventional biomarkers of immunosuppression, the combination of high sPD-L1 and low mHLA-DR levels at D5–8 identified a subgroup of severely ill patients with particularly unfavorable outcomes. These promising findings should be validated in additional cohorts covering a broader range of sepsis severity. A deeper understanding of the role of sPD-L1 in sepsis could help position it within a panel of biomarkers aimed at guiding immunomodulatory therapies in cases of persistent sepsis-induced immunosuppression.

## CRediT authorship contribution statement

All authors contributed to the analysis and interpretation of data and to critical revision of the manuscript for important intellectual content. CB performed the statistical analyses and drafted the initial manuscript. GM, ACL, and FV contributed substantially to the study conception and design. TL built the clinical database and supervised the statistical analyses. ACL was responsible for patient inclusion and supervision of clinical data acquisition. CB, EM, and APF generated the biological data. All authors reviewed and edited the manuscript and approved the final submitted version.

## Consent for publication

Not applicable.

## Ethics approval and consent to participate

The patients belonged to the IMMUNOSEPSIS study registered at clinicaltrials.gov (NCT04067674). Because of the non interventional nature of the study, written informed consent was not required for inclusion. However, each clinician orally informed the patient or family members about the objectives, methodology, and conduct of the study, and provided them with a leaflet using plain language. Non opposition to participation to this study was systematically obtained from the patient or a third party before any blood sampling was performed and was recorded in patient’s clinical file. All procedures performed were in accordance with the ethical standards of the institutional and/or national research committee (Comité de Protection des Personnes Ouest II, IRB number #19.01.23.71957) and with the 1964 Helsinki declaration and its later amendments or comparable ethical standards.

## Funding

The present study was supported by funds from Hospices Civils de Lyon (AO 2024 Jeune Chercheur PAM Biologie et Anatomie Pathologique to EM and APF) and intra-mural funding of EA7426 (Lyon 1 University).

## Availability of data and material

The original contributions presented in the study are included in the article. Further inquiries can be directed to the corresponding author.

## Declaration of competing interest

The authors declare they have no conflict of interest.
